# Influence of social support and coping strategies on psychological stress among frontline medical personnel during the Yangbi Earthquake: a cross-sectional analysis

**DOI:** 10.3389/fpsyt.2024.1473005

**Published:** 2024-09-27

**Authors:** Jiafeng Li, Jing Ye, Xiaolan Yang, Huan Sun, Hui Yan, Yiwen Yuan, Yang Peng, Xiangdong Tang

**Affiliations:** ^1^ Mental Health Center, West China Hospital of Sichuan University, Chengdu, China; ^2^ Sleep Medicine Center, The First People’s Hospital of Yunnan, Kunming, China; ^3^ Department of Psychiatry, West China Second University Hospital, Sichuan University, Chengdu, China; ^4^ Emergency Office, West China Hospital of Sichuan University, Chengdu, China; ^5^ Department of Psychosomatic Medicine, Chengdu Fifth People’s Hospital, Chengdu, China; ^6^ President’s Office, West China Hospital of Sichuan University, Chengdu, China; ^7^ Sleep Medicine Center, West China Hospital of Sichuan University, Chengdu, China; ^8^ Department of Respiratory and Critical Care Medicine, West China Hospital of Sichuan University, Chengdu, China

**Keywords:** earthquake, frontline medical staff, psychological stress, coping strategies, social support

## Abstract

**Objectives:**

This study aimed to investigate the psychological stress experienced by frontline medical staff during the Yangbi Earthquake and to understand how coping strategies and social support influence stress responses.

**Methods:**

From days 3 to 14 post-earthquake, online questionnaires were administered to frontline medical staff to assess perceived social support, coping strategies, and psychological stress responses using the Perceived Social Support Scale (PSSS), Trait Coping Strategies Questionnaire (TCSQ), and Stress Response Questionnaire (SRQ). Data analysis included correlation analysis to explore relationships between variables, multiple linear regression to identify key predictors of stress, and path analysis to determine direct and indirect effects.

**Results:**

A total of 253 valid questionnaires were analyzed, with a participant composition of 81.82% females and 18.18% males, and the majority being nurses (62.06%). Psychological stress responses varied by gender and age, with females and older age groups showing higher physical stress responses (P < 0.05). Correlation and regression analyses indicated that negative coping and lower levels of social support were associated with increased stress responses (P < 0.05). Path analysis revealed that intra-family and extra-family support influenced stress responses directly and indirectly through coping strategies (P < 0.05).

**Conclusion:**

This study suggests that perceived social support directly influences stress responses in frontline medical personnel during disasters, with coping strategies mediating this effect. Future research should explore these dynamics over time through longitudinal studies.

## 1 Introduction

Natural disasters, particularly earthquakes, often exert significant psychological pressure on affected individuals. In recent years, there has been an increasing recognition of the heightened psychological stress faced by healthcare workers during disaster scenarios, which not only impacts their mental and physical well-being but also their performance in emergency situations (1, 2). Despite this, most existing research has focused on the general population, leaving a notable gap in understanding the unique challenges encountered by healthcare workers, especially those operating in minority regions affected by disasters (3–7).

On May 21st, 2021, a magnitude 6.4 earthquake struck Yangbi in the Dali Bai Autonomous Prefecture of Yunnan Province, resulting in three fatalities and thirty-four injuries (8–11). Local emergency and medical departments were among the first to respond, with medical personnel receiving disaster relief instructions and immediately engaging in intensive rescue operations. These frontline medical staff faced prolonged periods of high-intensity work, which significantly elevated their psychological stress levels (12–14). Excessive stress in such environments can lead to negative emotions such as anxiety and depression, as well as physical reactions like sleep deprivation, and in severe cases, the development of acute stress disorder (ASD) (15–19).

Healthcare workers play a critical role in disaster relief, yet their psychological stress responses, social support and coping strategies, especially in the context of earthquake rescue operations in minority regions, are not well understood. Existing research often fails to account for the specific challenges faced by healthcare workers in areas like Yangbi, China. These workers may experience additional difficulties due to factors such as local practices, language differences, and varying support networks, all of which can affect their stress levels and coping strategies.

This study aims to address this gap by examining the psychological stress responses, social support and coping strategies of healthcare personnel involved in the earthquake rescue operations in Yangbi, Yunnan Province. We used correlation analysis and multiple linear regression to explore the relationships and predictors of stress levels, followed by path analysis to understand the direct and indirect effects among stress responses, social support, and coping strategies. Our hypothesis suggests that social support affects stress responses directly, with coping strategies serving as mediators. By focusing on these unique settings, this research provides insights into the psychological resilience of healthcare workers in minority regions and offers theoretical support for tailored psychological interventions in disaster scenarios.

## 2 Methods

### 2.1 Inclusion criteria

Participants were included in the study based on the following criteria: 1) Active frontline medical personnel involved in the earthquake relief efforts on May 21, 2021, which included clinical physicians, nurses, administrative staff, and logistical personnel; 2) Absence of any cognitive impairments, ensuring the ability to understand and independently complete the questionnaire; 3) Informed awareness of the study and voluntary participation; 4) Self-reported absence of any history of physical or mental illnesses at the time of participation.

### 2.2 Exclusion criteria

Participants were excluded if they self-reported a history of mental or psychological illnesses. This exclusion criterion was intended to ensure that the observed stress responses were directly related to the earthquake relief efforts rather than pre-existing conditions.

### 2.3 Research design

A cross-sectional study design was used. Data collection was conducted via an online survey distributed through the Wenjuanxing platform (https://www.wjx.cn) after the earthquake. The survey link was shared in work-related WeChat groups associated with clinical, nursing, and administrative departments involved in the relief efforts. Participation was encouraged through departmental announcements. The timeline for data collection was set from the 3rd to the 14th day post-earthquake to align with the acute stress response period, which is critical for capturing the immediate psychological impact on the frontline personnel. The survey was administered once within this period to evaluate the stress responses, perceived social support, and coping strategies following the disaster. Upon accessing the survey, participants were presented with an informed consent form. Only those who met the inclusion criteria and provided consent were allowed to proceed. The survey captured both demographic and psychometric data to comprehensively cover the study variables.

### 2.4 Data collection

#### 2.4.1 General information

This form collected demographic information such as gender, age, education level, job position, professional title, years of work experience, and the level of the hospital where participants were employed during the earthquake. This information was essential for analyzing the influence of sociodemographic variables on stress responses.

#### 2.4.2 Stress response questionnaire

The SRQ is a validated instrument designed to assess the physical and emotional symptoms that individuals experience in response to social stress. The questionnaire comprises 28 items, categorized into three subscales: Emotional Reactions, which captures the emotional effects of stress; Coping Reactions, which evaluates behavioral responses to stress; and Physiological Reactions, which measures physical symptoms associated with stress. Participants respond to each item on a 5-point Likert scale, where higher scores reflect more significant stress responses. The SRQ has been widely used for stress assessment in disaster contexts (50).

#### 2.4.3 Perceived social support scale

The PSSS measures perceived social support from family, friends, and significant others. The scale consists of 12 items rated on a 7-point Likert scale, with higher scores indicating greater perceived social support (20). This scale is widely used in stress and coping research, providing a robust measure of social support (21).

#### 2.4.4 Trait coping strategies questionnaire

The TCSQ evaluates stable coping strategies tied to personality traits, using 20 items rated on a 5-point Likert scale. It includes two subscales: Positive Coping, which measures adaptive strategies, and Negative Coping, which assesses maladaptive responses. Higher scores indicate greater reliance on the corresponding coping strategy, providing insight into individuals’ habitual stress responses (51).

### 2.5 Data analysis

Data analysis was conducted using R software version 4.0.3, employing a range of statistical methods. Descriptive statistics were used to summarize the demographic characteristics of the participants. Comparative analyses, including t-tests, ANOVA, and non-parametric tests, were performed to examine differences in stress responses, coping strategies, and perceived social support across demographic groups such as age, gender, and job title. Pearson correlation coefficients were calculated to explore the relationships among stress responses, coping strategies, and social support. Multiple linear regression was used to identify predictors of stress responses, adjusting for confounding factors like age and gender. To further assess and visualize both direct and indirect relationships among stress responses, coping strategies, and social support, path analysis using structural equation modeling was employed. To control for the risk of false positives due to multiple comparisons, we applied the Bonferroni correction, adjusting the p-values by dividing the significance level of each test by the number of comparisons. Statistical significance was set at α = 0.05.

### 2.6 Quality control

To ensure the quality and integrity of the data, the Wenjuanxing platform tracked IP addresses, allowing only one response per participant. The platform also monitored the time spent on each survey, excluding responses completed in less than 100 seconds to ensure data reliability. The survey was designed to protect participant confidentiality by not collecting sensitive personal information, which encouraged honest and accurate responses.

## 3 Results

### 3.1 Overview of study participants

A total of 264 questionnaires were distributed, and 253 valid responses were collected, yielding an effective response rate of 95.8%. Among the participants, 46 were male (18.18%) and 207 were female (81.82%), with ages ranging from 23 to 57 years. The participants included 47 doctors (18.58%), 157 nurses (62.06%), and 49 non-clinical staff (19.36%). Most held junior titles (65.22%), with smaller proportions holding intermediate (17.79%), associate senior (7.9%), and senior titles (1.98%). Regarding work experience, 18.97% had less than 3 years, 28.46% had 3-5 years, 21.74% had 6-10 years, and 30.83% had more than 10 years.

### 3.2 Stress response, social support, and coping strategies among local medical staff

The local medical staff involved in the rescue reported an average body response score of 17.80 ± 9.03 and a behavior response score of 10.49 ± 5.67. The total stress response score was 52.91 ± 25.39, with an emotional response score of 21.30 ± 10.87. In terms of social support, local medical staff reported intra-family support scores of 22.53 ± 5.59 and extra-family support scores of 42.35 ± 10.31, with a total social support score of 57.32 ± 12.38. Regarding coping strategies, local medical staff exhibited an average positive coping score of 27.54 ± 7.72 and a negative coping score of 35.35 ± 8.86. Detailed results are presented in **Table 1**.

**Table 1:** Stress response, social support, and coping strategies among local medical staff (Score, M ± SD).

Questionnaires	Local medical staff (M ± SD)
Stress response questionnaire (SRQ)	
Emotion Response	21.30 ± 10.87
Body Response	17.80 ± 9.03
Behavior Response	10.49 ± 5.67
Stress Response Score in Total	52.91 ± 25.39
Perceived social support scale (PSSS)	
Intra-Family Support	22.53 ± 5.59
Extra-Family Support	42.35 ± 10.31
Support score in Total	57.32 ± 12.38
Trait coping strategies questionnaire (TCSQ)	
Positive Coping	27.54 ± 7.72
Negative Coping	35.35 ± 8.86

### 3.3 Psychological stress response results

Statistical differences were found in physical reactions between different genders, with females exhibiting significantly higher levels of physical reactions than males (P = 0.02). No significant differences were observed in emotional reactions or behavioral reactions between genders. Across age groups, the 45-59 age group exhibited significantly higher physical reactions compared to others (P = 0.03), though emotional reactions, behavioral reactions, and total stress response scores did not differ significantly by age. Additionally, there were no significant differences in psychological stress responses among medical personnel based on professional titles or positions (P > 0.05). Significant differences in psychological stress responses were observed based on years of working experience. Those with 21-30 years of experience had the highest physical reaction scores (M = 24.88, SD = 12.06), as well as the highest scores for emotional reactions (P = 0.006) and behavioral reactions (P = 0.032). Overall, total stress response scores were also highest in the 21-30 years working experience group (P = 0.027). See **Table 2** for further details.

**Table 2:** Psychological stress response in rescue-involved local medical staff (M ± SD).

	Number (%)	Emotion response	Body response	Behavioral response	Stress response score in Total
Gender					
Male	46 (18.18)	20.09 ± 11.60	15.24 ± 7.96	10.33 ± 5.75	50.28 ± 28.20
Female	207 (81.82)	21.56 ± 10.71	18.37 ± 9.17	10.52 ± 5.67	52.74 ± 24.30
*t*		-0.793	-2.341	-0.209	-0.548
*P*		0.431	0.02*	0.834	0.585
Age					
18-29	123 (48.62)	20.89 ± 10.22	17.07 ± 8.67	9.99 ± 5.49	51.00 ± 24.01
30-44	100 (39.53)	20.68 ± 11.05	17.46 ± 8.91	10.65 ± 5.52	50.75 ± 24.95
45-59	30 (11.85)	25.03 ± 12.40	21.90 ± 10.10	11.97 ± 6.71	62.77 ± 27.51
*F*		1.89	4.71	2.920	3.041
*P*		0.17	0.03*	0.088	0.082
Occupation					
Doctor	47 (18.58)	21.51 ± 11.65	17.64 ± 9.10	10.82 ± 5.84	54.28 ± 29.09
Nurse	157 (62.06)	21.31 ± 10.78	17.87 ± 8.88	10.54 ± 5.70	51.79 ± 23.57
Non-medical staff	49 (19.36)	21.06 ± 10.61	17.73 ± 9.64	10.00 ± 5.51	52.02 ± 25.76
*F*		0.041	0.002	0.517	0.189
*P*		0.84	0.96	0.473	0.664
Professional title					
Junior	165 (65.22)	21.74 ± 11.11	18.05 ± 9.27	10.57 ± 5.64	52.66 ± 24.32
Intermediate	45 (17.79)	19.82 ± 9.91	16.8 ± 8.47	10.58 ± 5.98	47.93 ± 23.79
Associate Senior	20 (7.90)	23.15 ± 12.52	18.65 ± 8.71	11.20 ± 6.44	59.05 ± 28.63
Full Senior	5 (1.98)	24.60 ± 9.61	22.80 ± 10.69	11.20 ± 7.94	63.60 ± 26.08
None	18 (7.11)	17.94 ± 9.05	15.67 ± 8.25	8.50 ± 3.29	49.22 ± 29.60
*F*		0.84	0.286	0.905	0.002
*P*		0.36	0.593	0.342	0.962
Years of working experience					
0-3	48 (18.97)	19.81 ± 10.00	15.79 ± 7.71	9.23 ± 4.58	48.39 ± 21.49
3-5	72 (28.46)	20.25 ± 9.85	16.96 ± 8.41	9.94 ± 5.49	50.38 ± 24.65
6-10	55 (21.74)	22.31 ± 12.34	18.05 ± 9.73	11.38 ± 6.43	52.33 ± 26.70
11-20	40 (15.81)	20.63 ± 10.73	18.03 ± 9.91	10.30 ± 4.89	51.05 ± 25.13
21-30	33 (13.04)	24.88 ± 12.06	21.70 ± 9.73	12.33 ± 6.91	63.81 ± 27.36
31-40	5 (1.98)	21.20 ± 5.63	18.80 ± 2.49	9.80 ± 2.39	51.00 ± 8.51
*F*		3.281	7.606	4.651	4.983
*P*		0.07	0.006**	0.032*	0.027*

*P <0.05, ** P <0.01, ***P<0.001.

### 3.4 Correlation analysis

Single-factor correlation analysis revealed significant correlations between the psychological stress response of local medical personnel and factors such as positive coping, negative coping, intra-family support, external family support, and total social support (P < 0.05). After adjusting for gender and age-related differences in physical reactions, it was found that negative coping, intra-family support, external family support, and overall social support were negatively correlated with stress response. Detailed results are provided in **Table 3**.

**Table 3:** Results of correlation analysis.

	Emotion Response	Body Response	Behavioral Response	Stress Response Score in Total
Positive Coping	0.034	0.032	0.045	0.002
Negative Coping	-0.619***	-0.569***	-0.633***	-0.583***
Intra-Family Support	-0.187**	-0.140	-0.258***	-0.202**
Extra-Family Support	-0.233***	-0.184**	-0.318***	-0.256***
Social Support	-0.252***	-0.197**	-0.330***	-0.271***

*P <0.05, ** P <0.01, ***P<0.001.

### 3.5 Regression analysis of psychological stress response

Multiple linear regression analysis was conducted to examine the effects of positive coping, negative coping, intra-family support, and external family support on the stress response among local medical personnel involved in rescue efforts. The regression model was statistically significant (P < 0.001) and explained 96.8% of the variance in stress response (adjusted R²= 0.968). After adjusting for age and gender, the analysis showed that coping strategies and social support significantly influence psychological stress responses. Positive coping had a standardized coefficient (β) of 0.24 (95% CI: 0.20, 0.27, p < 0.001), while negative coping had a β of 0.09 (95% CI: 0.06, 0.12, p < 0.001). Intra-family support had a strong positive association with stress responses (β = 0.69, 95% CI: 0.59, 0.78, p < 0.001), and extra-family support had the strongest positive association (β = 0.82, 95% CI: 0.77, 0.87, p < 0.001) (**Table 4**).

**Table 4:** Regression analysis results.

	*β*	95%CI	Adjusted *P*
Positive Coping	0.24	(0.20, 0.27)	<0.001
Negative Coping	0.09	(0.06, 0.12)	<0.001
Intra-Family Support	0.69	(0.59, 0.78)	<0.001
Extra-Family Support	0.82	(0.77, 0.87)	<0.001

### 3.6 Path analysis of psychological stress response

**Figure 1:**
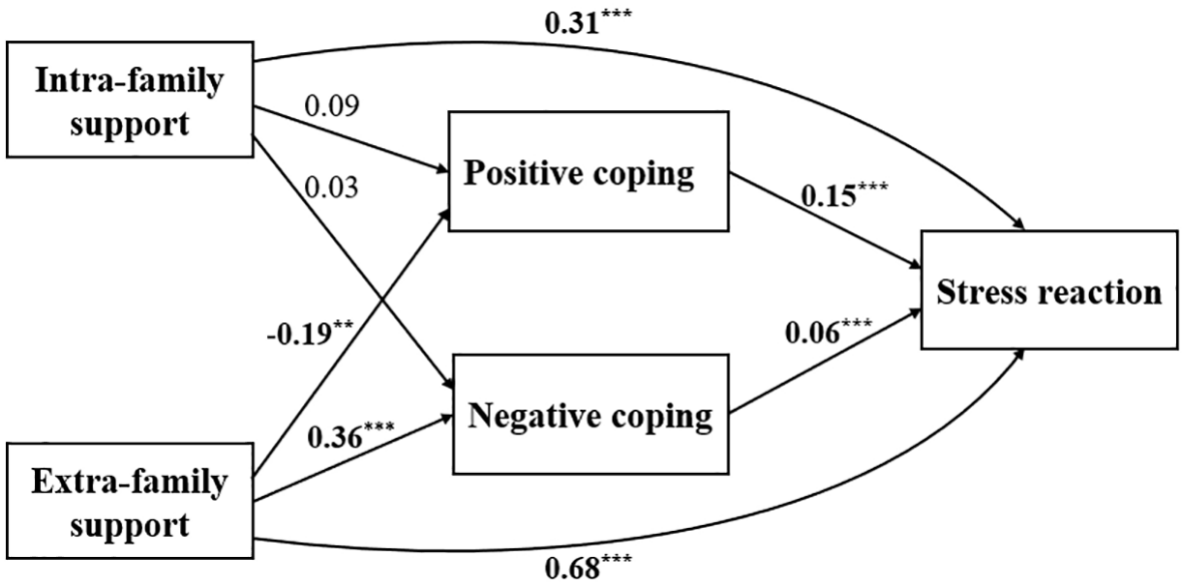
Path diagram of the relationship between coping strategies, social support, and psychological stress among local frontline medical staff. *P <0.05, ** *P* <0.01, ***P<0.001.

A path analysis was conducted to examine the direct and indirect effects of intra-family support (X1), external family support (X2), positive coping (X3), and negative coping (X4) on the psychological stress response among frontline medical personnel involved in local rescue efforts. The path diagram (**Figure 1**) and corresponding coefficients (**Table 5**) show that intra-family support, external family support, positive coping, and negative coping directly impact stress response, with coefficients of 0.31, 0.68, 0.15, and 0.06, respectively. Additionally, intra-family and external family support indirectly affect stress response through positive and negative coping strategies, with indirect effects of 0.0153 and -0.0069, respectively. These findings suggest that both intra-family and external family support influence psychological stress directly and indirectly by shaping coping strategies, which mediate the relationship between support systems and stress responses.

**Table 5:** Results of path model effect analysis.

Independent variable	Direct effect	Indirect effect	Total effect
Intra-Family Support(X1)	0.31	X1→X3→Y, X1→X4→Y 0.09×0.15 + 0.03×0.06 = 0.0153	0.3253
Extra-Family Support(X2)	0.68	X2→X3→Y, X2→X4→Y -0.19×0.15 + 0.36×0.06= -0.0069	0.6731
Positive Coping(X3)	0.15	0	0.15
Negative Coping(X4)	0.06	0	0.06

## 4 Discussion

This study highlights the significant impact of coping strategies and social support on the psychological stress responses of frontline medical personnel involved in local rescue efforts. The results underscore the importance of positive coping strategies and strong social support in mitigating stress and enhancing overall well-being.

Consistent with previous studies, we observed that higher levels of social support are associated with better mental health outcomes among healthcare workers (22–24). Our study further confirms that local medical staff involved in earthquake rescue operations experience significantly higher stress compared to the general population (20). This finding is in line with broader research from disaster contexts, such as the COVID-19 pandemic, where healthcare workers consistently report elevated stress levels. (6, 25, 26).

Gender differences were evident, with female medical staff reporting higher physical stress levels, likely due to different coping strategies, support networks, and physiological stress responses (27–29). This suggests the need for gender-sensitive interventions tailored to female healthcare workers’ unique stressors (30). Age-related differences also emerged, with healthcare workers aged 45-59 showing higher stress, potentially due to increased responsibilities or age-related health concerns (31–33). Age-specific support strategies could mitigate these stress responses effectively.

Our analysis revealed that work experience significantly impacts stress responses, with workers having 21-30 years of experience reporting the highest stress levels. This could be due to cumulative stress or perceived workload (34, 35). These findings highlight the need to consider work experience when designing support systems and interventions. Tailored strategies, such as peer support programs (36), could help more experienced personnel manage stress more effectively while leveraging their experience to support less experienced colleagues.

Both positive and negative coping strategies were found to impact stress responses, with positive coping having a more substantial effect (β = 0.24) compared to negative coping (β = 0.09). The negative correlation between negative coping and stress suggests that strong social support can buffer stress, even when maladaptive coping strategies are used (37). This highlights the importance of a robust support network in managing stress, even in high-pressure disaster settings.

Our analysis reveals that the paths of influence identified align with the existing literature, emphasizing the crucial role of social support in stress response (38–40). Our findings confirm that social support, particularly from external family sources, has direct effects on stress levels, while coping strategies serve as important mediators in this relationship (41, 42). Specifically, external family support emerged as the most significant direct factor influencing stress response, consistent with previous studies highlighting the importance of social support networks, especially in disaster contexts (43, 44).

Moreover, the mediation role of coping strategies, which enhances the benefits of social support, is well-supported by existing literature (45). This is particularly relevant in professional settings, where external support networks can play a crucial role in mitigating work-related stress (46, 47). Notably, our findings suggest that while both internal and external family support are important, the influence of external family support is more significant, potentially due to the unique stressors faced by our participants. This observation underscores the importance of considering the specific characteristics of the study population and the context when interpreting the relative importance of different support systems.

Interestingly, our results also indicate that social support, especially from external sources, is associated with increased reporting of stress. This complexity highlights that while social support does not always reduce stress, it significantly influences how stress is managed and reported (4). Furthermore, reliance on negative coping strategies, even with strong external family support, may increase anxiety levels (48). This finding emphasizes the complexity of the relationship between coping strategies and social support and underscores the need for interventions that are multidimensional and tailored to the specific context of the population at risk (49).

Despite its insights, our study has several limitations. First, the cross-sectional design prevents us from inferring causality between social support, coping strategies, and stress responses. Second, collecting data only once, between days 3 and 14 post-earthquake, restricts our ability to capture fluctuations in stress, support, and coping over time. Third, the small sample size and reliance on self-reported measures may affect the generalizability of our findings and introduce response biases. Additionally, we did not account for confounding factors, such as pre-existing trauma or mental health conditions, which could have influenced the results. Future research should use longitudinal designs with larger, more diverse samples and objective measures to better understand how stress and coping mechanisms evolve over time and in different contexts.

## 5 Conclusion

This study shows that perceived social support directly impacts stress responses in frontline medical personnel during disasters, with coping strategies serving as mediators. Factors like gender, age, and work experience also significantly affect stress levels, indicating the need for targeted interventions. Strengthening support networks and promoting positive coping strategies are essential for reducing stress. Future research should investigate the long-term dynamics of stress and coping through longitudinal studies.

## Data Availability

The raw data supporting the conclusions of this article will be made available by the authors, without undue reservation.

## References

[1] Ding X Jian Z Xu Y Lin Z Chen Z Zhang Y. Psychological stress and coping strategies among frontline healthcare workers supporting patients with coronavirus disease 2019: a retrospective study and literature review. Ther Adv Respir Dis. (2022) 16:17534666221130215. doi: 10.1177/17534666221130215 36476064 PMC9742697

[2] Platania S Gruttadauria SV Morando M. Dispositional resilience as mediator in psychological stress on healthcare workers: A multi-group analysis of frontline and non-frontline workers. Eur J Investig Health Psychol Educ. (2022) 12:1285–99. doi: 10.3390/ejihpe12090089 PMC9497834 36135227

[3] Adhikari Baral I K CB. Post traumatic stress disorder and coping strategies among adult survivors of earthquake, Nepal. BMC Psychiatry. (2019) 19:118. doi: 10.1186/s12888-019-2090-y 30999893 PMC6474064

[4] Hong C Cao J Efferth T. Posttraumatic stress disorder among earthquake survivors of the Wenchuan area (Sichuan, China). Eur J Psychotraumatol. (2014) 5:26531. doi: 10.3402/ejpt.v5.26531 25511735 PMC4265177

[5] Hong C Efferth T. Systematic review on post-traumatic stress disorder among survivors of the Wenchuan earthquake. Trauma Violence Abuse. (2016) 17:542–61. doi: 10.1177/1524838015585313 26028651

[6] Luan R Pu W Dai L Yang R Wang P. Comparison of psychological stress levels and associated factors among healthcare workers, frontline workers, and the general public during the novel coronavirus pandemic. Front Psychiatry. (2020) 11:583971. doi: 10.3389/fpsyt.2020.583971 33335490 PMC7736032

[7] Xu J Wang Y Tang W. Posttraumatic stress disorder in Longmenshan adolescents at three years after the 2013 Lushan earthquake. Gen Hosp Psychiatry. (2018) 54:45–51. doi: 10.1016/j.genhosppsych.2018.05.009 29861052

[8] Cao Y Li Y Li Z Cao K. Rapid assessment of disasters caused by Yangbi M 6.4 earthquake in Yunnan Province. China Earthquake Eng J. (2021) 43:751–9. doi: 10.3969/j.issn.1000-0844.2021.04.751

[9] Duan M Zuo K Zhao C Zhou L. Seismogenic environment and mechanism of the Yangbi MS6.4 earthquake in Yunnan, China. Earthquake Sci. (2022) 35:297–310. doi: 10.1016/j.eqs.2022.08.001

[10] Liang S Guo R Yang H Tang X Xu X Gan W. Rupture imaging of the 2021 Ms 6.4 Yangbi, China, earthquake: Implications for the diffuse deformation in the northern region of the Red River fault. Tectonophysics. (2023) 862:229932. doi: 10.1016/j.tecto.2023.229932

[11] Zhu G Yang H Tan YJ Jin M Li X Yang W. The cascading foreshock sequence of the Ms 6.4 Yangbi earthquake in Yunnan, China. Earth Planet Sci Lett. (2022) 591:117594. doi: 10.1016/j.epsl.2022.117594

[12] Fenollar F Bouam A Ballouche M Fuster L Prudent E Colson P. Evaluation of the panbio COVID-19 rapid antigen detection test device for the screening of patients with COVID-19. J Clin Microbiol. (2021) 59. doi: 10.1128/jcm.02589-20 PMC8111145 33139420

[13] Ruotsalainen JH Verbeek JH Mariné A Serra C. Preventing occupational stress in healthcare workers. Cochrane Database Syst Rev. (2014) 11):Cd002892. doi: 10.1002/14651858.CD002892.pub3 25391582

[14] Song Y Lindquist R. Effects of mindfulness-based stress reduction on depression, anxiety, stress and mindfulness in Korean nursing students. Nurse Educ Today. (2015) 35:86–90. doi: 10.1016/j.nedt.2014.06.010 25066651

[15] Cahill SP Pontoski K. Post-traumatic stress disorder and acute stress disorder I: their nature and assessment considerations. Psychiatry (Edgmont). (2005) 2:14–25. PMC3004735 21179648

[16] Chang Q Su H Xia Y Gao S Zhang M Ma X. Association between clinical competencies and mental health symptoms among frontline medical staff during the COVID-19 outbreak: A cross-sectional study. Front Psychiatry. (2022) 13:760521. doi: 10.3389/fpsyt.2022.760521 35558425 PMC9086962

[17] Li L Wang X Tan J Li J Yuan Y. Influence of sleep difficulty on post-traumatic stress symptoms among frontline medical staff during COVID-19 pandemic in China. Psychol Health Med. (2022) 27:1924–36. doi: 10.1080/13548506.2021.1981411 34541987

[18] Visser E Gosens T Den Oudsten BL De Vries J. The course, prediction, and treatment of acute and posttraumatic stress in trauma patients: A systematic review. J Trauma Acute Care Surg. (2017) 82:1158–83. doi: 10.1097/ta.0000000000001447 28520689

[19] Zhang Z Hu Y Chen Y Liao Z Zheng Y Ding L. Sleep disorders and related factors among frontline medical staff supporting Wuhan during the COVID-19 outbreak. Bull Menninger Clin. (2021), 1–17. doi: 10.1521/bumc_2021_85_01 33939498

[20] Dahlem NW Zimet GD Walker RR. The Multidimensional Scale of Perceived Social Support: a confirmation study. J Clin Psychol. (1991) 47:756–61. doi: 10.1002/1097-4679(199111)47:6<756::aid-jclp2270470605>3.0.co;2-l 1757578

[21] Tonsing K Zimet GD Tse S. Assessing social support among South Asians: the multidimensional scale of perceived social support. Asian J Psychiatr. (2012) 5:164–8. doi: 10.1016/j.ajp.2012.02.012 22813661

[22] Ab Aziz WA Musa KI Ibrahim MI Osman Y Shafei MN. An association between job stress and poor social support among healthcare workers in Northeastern Malaysia. Cureus. (2023) 15:e38937. doi: 10.7759/cureus.38937 37313064 PMC10259192

[23] Huang J Liu Q Li J Li X You J Zhang L. Post-traumatic stress disorder status in a rescue group after the Wenchuan earthquake relief. Neural Regener Res. (2013) 8:1898–906. doi: 10.3969/j.issn.1673-5374.2013.20.009 PMC4145976 25206499

[24] Zhang D Li X Zhang M Huang A Yang L Wang C. The mediating effect of resilience and COVID-19 anxiety on the relationship between social support and insomnia among healthcare workers: a cross-sectional study. Front Psychiatry. (2024) 15:1328226. doi: 10.3389/fpsyt.2024.1328226 38414504 PMC10896830

[25] Liang Y Wu K Zhou Y Huang X Zhou Y Liu Z. Mental health in frontline medical workers during the 2019 novel coronavirus disease epidemic in China: A comparison with the general population. Int J Environ Res Public Health. (2020) 17(18):6550. doi: 10.3390/ijerph17186550 32916836 PMC7558595

[26] Stoyanov D Stoyanova K. Commentary: coronavirus disease 2019 worry and related factors: Turkish adaptation and psychometric properties of the coronavirus disease 2019 worry scale. Alpha Psychiatry. (2023) 24:77–8. doi: 10.5152/alphapsychiatry.2023.310123 PMC10151968 37144052

[27] Goldfarb EV Seo D Sinha R. Sex differences in neural stress responses and correlation with subjective stress and stress regulation. Neurobiol Stress. (2019) 11:100177. doi: 10.1016/j.ynstr.2019.100177 31304198 PMC6603439

[28] Passarelli M Casetta L Rizzi L Perrella R. Responses to stress: investigating the role of gender, social relationships, and touch avoidance in Italy. Int J Environ Res Public Health. (2021) 18(2):600. doi: 10.3390/ijerph18020600 33445696 PMC7828124

[29] Suitor J Gilligan M. How gender shapes patterns and consequences of support in later life. Innovation Aging. (2018) 2:344–4. doi: 10.1093/geroni/igy023.1263

[30] Hamama-Raz Y Palgi Y Shrira A Goodwin R Kaniasty K Ben-Ezra M. Gender differences in psychological reactions to Hurricane Sandy among New York Metropolitan Area residents. Psychiatr Q. (2015) 86:285–96. doi: 10.1007/s11126-014-9333-3 25428781

[31] Bleil ME Adler NE Pasch LA Sternfeld B Gregorich SE Rosen MP. Psychological stress and reproductive aging among pre-menopausal women. Hum Reprod. (2012) 27:2720–8. doi: 10.1093/humrep/des214 PMC3415289 22767452

[32] Choi W Lee SJ Lee WJ Beak EM Kim KY. Job satisfaction level of safety and health manager in construction industry: pandemic period. Int J Environ Res Public Health. (2022) 19(2):831–42. doi: 10.3390/ijerph19105858 PMC9141010 35627394

[33] Grover S Sahoo S Dua D Mehra A Nehra R. Psychological impact of COVID-19 duties during lockdown on police personnel and their perception about the behavior of the people: an exploratory study from India. Int J Ment Health Addict. (2022) 20:831–42. doi: 10.1007/s11469-020-00408-8 PMC7643718 33173448

[34] Cheng Z Tao Y Liu T He S Chen Y Sun L. Psychology, stress, insomnia, and resilience of medical staff in China during the COVID-19 policy opening: a cross-sectional survey. Front Public Health. (2023) 11:1249255. doi: 10.3389/fpubh.2023.1249255 37693701 PMC10485264

[35] Li M Xia L Yang Y Zhang L Zhang S Liu T. Depression, anxiety, stress, and their associations with quality of life in a nationwide sample of psychiatrists in China during the COVID-19 pandemic. Front Psychol. (2022) 13:881408. doi: 10.3389/fpsyg.2022.881408 35814128 PMC9260312

[36] Chan AO Kee JP Chan YH. Awareness and utilization of peer support programs in Singapore public general hospitals. Int J Emerg Ment Health. (2012) 14:217–23. 23894802

[37] Liu D Cui Z Zhang Q Liu F Chen H Wang J. The mediating role of specific coping styles in the relationship between perceived social support and depressive symptoms in adolescents. J Affect Disord. (2023) 325:647–55. doi: 10.1016/j.jad.2023.01.043 36669570

[38] Robinson H Ravikulan A Nater UM Skoluda N Jarrett P Broadbent E. The role of social closeness during tape stripping to facilitate skin barrier recovery: Preliminary findings. Health Psychol. (2017) 36:619–29. doi: 10.1037/hea0000492 28277705

[39] Rockliff HE Lightman SL Rhidian E Buchanan H Gordon U Vedhara K. A systematic review of psychosocial factors associated with emotional adjustment in *in vitro* fertilization patients. Hum Reprod Update. (2014) 20:594–613. doi: 10.1093/humupd/dmu010 24676468

[40] Zhu D He Y Wang F Li Y Wen X Tong Y. Inconsistency in psychological resilience and social support with mental health in early adolescents: A multilevel response surface analysis approach. J Affect Disord. (2024) 361:627–36. doi: 10.1016/j.jad.2024.06.086 38925311

[41] Braun-Lewensohn O Sagy S Roth G. Coping strategies as mediators of the relationship between sense of coherence and stress reactions: Israeli adolescents under missile attacks. Anxiety Stress Coping. (2011) 24:327–41. doi: 10.1080/10615806.2010.494329 20582754

[42] Thompson NJ Fiorillo D Rothbaum BO Ressler KJ Michopoulos V. Coping strategies as mediators in relation to resilience and posttraumatic stress disorder. J Affect Disord. (2018) 225:153–9. doi: 10.1016/j.jad.2017.08.049 PMC5626644 28837948

[43] Liao C Guo L Zhang C Zhang M Jiang W Zhong Y. Emergency stress management among nurses: A lesson from the COVID-19 outbreak in China-a cross-sectional study. J Clin Nurs. (2021) 30:433–42. doi: 10.1111/jocn.15553 33141483

[44] Tselebis A Lekka D Sikaras C Tsomaka E Tassopoulos A Ilias I. Insomnia, Perceived Stress, and Family Support among Nursing Staff during the Pandemic Crisis. Healthc (Basel). (2020) 8(4):434. doi: 10.3390/healthcare8040434 PMC7712233 33114662

[45] Adekanmbi FP Ukpere WI Kelvin-Iloafu LE. The relational effects of perceived organizational support, fear of COVID-19, and work-related stress on the safety performance of healthcare workers. Front Psychol. (2022) 13:963683. doi: 10.3389/fpsyg.2022.963683 36300066 PMC9588951

[46] Engels M Scheepers L Engels J Boß L Kuhlmann R Kuske J. Web-based occupational stress prevention in German micro- and small-sized enterprises - process evaluation results of an implementation study. BMC Public Health. (2024) 24:1618. doi: 10.1186/s12889-024-19102-8 38886711 PMC11184923

[47] Sablone S Groicher M Patrizia Fanco T Risola R G MV Bellino M. Work-related stress amongst legal medical doctors: the need for systematic psychological support. An Italian perspective. Forensic Sci Res. (2023) 8:116–22. doi: 10.1093/fsr/owad018 PMC10445592 37621454

[48] Jankowska-Polańska B Polański J Chabowski M Rosińczuk J Mazur G. Influence of coping strategy on perception of anxiety and depression in patients with non-small cell lung cancer. Adv Exp Med Biol. (2020) 1251:57–70. doi: 10.1007/5584_2019_448 31802442

[49] Wang Y Wang P. Perceived stress and psychological distress among chinese physicians: The mediating role of coping style. Med (Baltimore). (2019) 98:e15950. doi: 10.1097/md.0000000000015950 PMC6571215 31169719

[50] Zhong X Jiang Q Qian L Wu Z. Correlation Between Stress Reaction and Social Support,Life Events, Coping Style in Medical Personnel. Chinese J Clin Psychol. (2005) 13(1):70–72. doi: 10.3969/j.issn.1005-3611.2005.01.025

[51] Jiang Q Zhu Y. Further explorations for a coping style questionnaire. Chinese J Behavior Med Sci. (1999) 8(3):167–169. doi: 10.3760/cma.j.issn.1674-6554.1999.03.003

